# Supplementary material for: Saccharomyces boulardii (CNCM I-745) improves intestinal damage in sepsis by remodeling intestinal flora structure
Source: Front Cell Infect Microbiol. 2025 Jul 31;15:1602792. doi: 10.3389/fcimb.2025.1602792 (PMC12350329; doi:10.3389/fcimb.2025.1602792)
Supplement: Supplementary file 1 [file DataSheet1.zip › summary/summary/6_Taxonomy_community/1_Taxonomy_profiling/4_Order/All/Order_abund_top30_sample.heatmap.pdf]

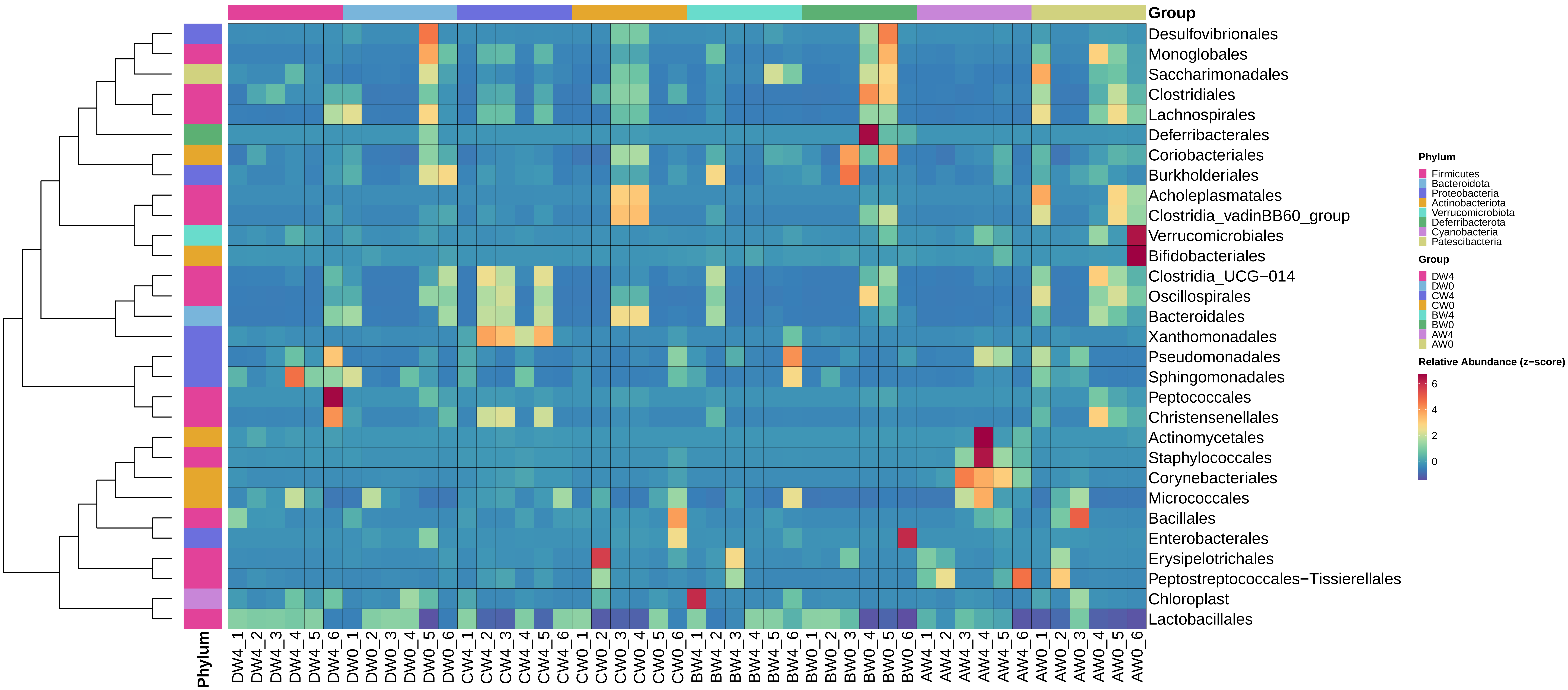

Group
Desulfovibrionales
Monoglobales
Saccharimonadales
Clostridiales
Lachnospirales
Deferribacterales
Coriobacteriales
Burkholderiales
Acholeplasmatales
Clostridia_vadinBB60_group
Verrucomicrobiales
Bifidobacteriales
Clostridia_UCG−014
Oscillospirales
Bacteroidales
Xanthomonadales
Pseudomonadales
Sphingomonadales
Peptococcales
Christensenellales
Actinomycetales
Staphylococcales
Corynebacteriales
Micrococcales
Bacillales
Enterobacterales
Erysipelotrichales
Peptostreptococcales−Tissierellales
Chloroplast
Lactobacillales
Phylum
DW4_1 DW4_2 DW4_3 DW4_4 DW4_5 DW4_6 DW0_1 DW0_2 DW0_3 DW0_4 DW0_5 DW0_6 CW4_1 CW4_2 CW4_3 CW4_4 CW4_5 CW4_6 CW0_1 CW0_2 CW0_3 CW0_4 CW0_5 CW0_6 BW4_1 BW4_2 BW4_3 BW4_4 BW4_5 BW4_6 BW0_1 BW0_2 BW0_3 BW0_4 BW0_5 BW0_6 AW4_1 AW4_2 AW4_3 AW4_4 AW4_5 AW4_6 AW0_1 AW0_2 AW0_3 AW0_4 AW0_5 AW0_6
Phylum
Firmicutes
Bacteroidota
Proteobacteria
Actinobacteriota
Verrucomicrobiota
Deferribacterota
Cyanobacteria
Patescibacteria
Group
DW4
DW0
CW4
CW0
BW4
BW0
AW4
AW0
Relative Abundance (z−score)
6
4
2
0
